# Enhancing Social and Emotional Wellbeing of Aboriginal Boarding Students: Evaluation of a Social and Emotional Learning Pilot Program

**DOI:** 10.3390/ijerph17030771

**Published:** 2020-01-26

**Authors:** Linél Franck, Richard Midford, Helen Cahill, Petra T. Buergelt, Gary Robinson, Bernard Leckning, Douglas Paton

**Affiliations:** 1 Psychology Department, College of Health and Human Sciences, Charles Darwin University, Darwin 0810, Australia; lfranck@bigpond.com (L.F.); douglas.paton@cdu.edu.au (D.P.); 2 Perth Psychological Services, National Drug Research Institute, Curtin University, Perth 6102, Australia; 3 Youth Research Centre, Graduate School of Education, University of Melbourne, Melbourne 3010, Australia; h.cahill@unimelb.edu.au; 4 School of Health Sciences, Faculty of Health, University of Canberra, Canberra 2617, Australia; petra.buergelt@canberra.edu; 5 Menzies School of Health Research, Centre for Child Development and Education, Darwin 0810, Australia; gary.robinson@menzies.edu.au (G.R.); Bernard.leckning@menzies.edu.au (B.L.)

**Keywords:** Aboriginal peoples, social and emotional learning, students, boarding school, Australia

## Abstract

Boarding schools can provide quality secondary education for Aboriginal students from remote Aboriginal Australian communities. However, transition into boarding school is commonly challenging for Aboriginal students as they need to negotiate unfamiliar cultural, social and learning environments whilst being separated from family and community support. Accordingly, it is critical for boarding schools to provide programs that enhance the social and emotional skills needed to meet the challenges. This study evaluated a 10-session social and emotional learning (SEL) program for Aboriginal boarders and identified contextual factors influencing its effectiveness. The study combined a pre-post quantitative evaluation using diverse social and emotional wellbeing measures with 28 students between 13–15 years (10 female, 11 male, 7 unidentified) and qualitative post focus groups with 10 students and episodic interviews with four staff delivering the program. Students’ social and emotional skills significantly improved. The qualitative findings revealed improvements in students seeking and giving help, working in groups, managing conflict, being assertive and discussing cultural issues. The focus groups and interviews also identified program elements that worked best and that need improvement. Secure relationships with staff delivering the program and participation in single sex groups stood out as critical enablers. The findings lend evidence to the critical importance of collaborative design, provision and evaluation of SEL programs with Aboriginal peoples.

## 1. Introduction

Schools are increasingly urged to play an active role in promoting the social and emotional wellbeing of their students and to pay particular attention to those most vulnerable to negative health and learning outcomes [1]. The secondary education system is seen as an important agent of change for Aboriginal Australians, with more young Aboriginal people participating in senior secondary education [2]. Boarding schools are seen as a way of providing high quality secondary education, particularly for students from remote communities. Accordingly, boarding schools are promoted in strategies to ‘close the gap’ of Aboriginal disadvantage, resulting in the number of Indigenous boarders increasing [3,4].

This education passage for Aboriginal boarders is, however, doubly fraught, because they are separated from the social and cultural support of their family and community, while at the same time having to adapt to a new cultural, social and learning environment and increased academic demands [5]. Consequently, it is important to build the capacity of boarding schools to provide programs that equip Aboriginal students with the social and emotional skills required for successful passage through secondary school and into adulthood. The important role of social and emotional learning (SEL) in the education process is recognized within the Close the Gap campaign with the Steering Committee stating: ‘promoting social and emotional wellbeing and resilience should also contribute to improving school attendance and performance because it will support children to cope with bullying and racism’ [6] (p. 39).

In addition, the wellbeing of young Aboriginal Australians is substantially worse than their non-Aboriginal peers. To address this situation, the Australian Government [7] declared enhancing the wellbeing of Indigenous peoples a priority in the National Aboriginal and Torres Strait Islander Health Plan. Poor social and emotional wellbeing (SEWB), and disproportionately high rates of mental health problems in this group, are the result of high levels of grief, intergenerational trauma and loss associated with colonization, frequent serious life stressors, and the ongoing deleterious effects of socioeconomic disadvantage and racism [8].

The Western Australian Aboriginal Child Health Survey (WAACHS) found that almost a quarter of Aboriginal children in Western Australia, aged 4–17 years, were at high risk of experiencing clinically significant emotional or behavioral difficulties—a proportion that was substantially higher than the state’s general child population [9]. Similarly, a national report on the wellbeing of Australian young people indicated that 33% of Australian Aboriginal young people, 18–24 years of age, reported high or very high levels of psychological distress, compared with 14% of their non-Aboriginal peers [10]. The consequence of low levels of SEWB for Aboriginal Australian young people, especially males, is a heightened mental health risk, which is associated with a range of poor outcomes. These outcomes include problematic alcohol and other drug use, high incarceration rates, self-harm and suicide [11,12,13,14].

This range of evidence as to the burden of social and emotional difficulties affecting Australian Aboriginal young people highlights an urgent need for effective, preventive efforts to reduce this disproportionate level of distress, increase educational success and improve life trajectories. Here, social and emotional learning (SEL) programs provide a promising pathway. SEL programs have been effective in increasing resilience to adversity, improving mental health and reducing suicidal ideation [7,15,16,17]. Research also indicates that SEL programs enhance social competence and academic success [18,19]. An obvious location for offering SEL programs are schools. Schools are places of learning and are well placed to facilitate the social and emotional development of their students through focused skill development programs, general teaching practices and the construction of a positive and inclusive school climate [19].

The benefit of schools formally teaching social and emotional skills is supported by an extensive literature. The OECD [1] published a comprehensive review of social and emotional learning, which indicates that raising levels of social and emotional skills, such as perseverance, self-esteem and sociability, can have beneficial effects on subjective wellbeing and health-related outcomes, as well as reducing anti-social behaviors. Social and emotional skills also interact with and potentiate cognitive skills to improve academic performance and enhance children’s likelihood of achieving positive outcomes in later life. The OECD [1] made a strong case for schools to emphasize development of the ‘whole child’, with a balanced set of cognitive, social and emotional skills, so that they are better equipped to face life’s challenges.

In their meta-analysis of programs that sought to enhance students’ social and emotional learning, Durlak and colleagues [19] identified that the most effective programs provided lessons that were sequenced, used active learning strategies, devoted sufficient time to skill development and had explicit learning goals. Their meta-analysis indicated that students receiving such programs felt more connected to their school and improved on measures of positive behavior, such as classroom discipline and attendance. Other studies found that students who participated in SEL programs were less likely to engage in anti-social behavior, such as bullying, fighting and problematic substance use, and displayed fewer mental health problems, such as depression, anxiety and alienation [20,21]. In terms of academic performance, when schools offered SEL programs, the achievement scores of their students improved by approximately 11% [19].

SEL programs have now been researched in developed and developing countries across a range of socioeconomic contexts and cultures, using both quantitative and qualitataive methods. The quantitative studies have included meta-analyses and randomized controlled trials that have investigated short-, medium- and long-term impacts of SEL [19,22]. These large-scale quantitative studies demonstrate that culturally responsive and evidence informed SEL programs can have significant long-term impacts on behavior, health and academic outcomes in urban and remote/rural settings, in ethnically homogenous or culturally diverse cohorts, regardless of family or guardian income.

Despite this large body of research, consideration of cultural responsiveness in the design of SEL programs is a relatively new area, with debate emerging about how social and emotional skills are defined, valued and expressed in different cultural and familial contexts. These contexts are important because cultural traditions shape norms of expression in relation to gender, age and context and also influence how emotions are expressed and interpreted [13,23,24]. Learners within collectivist cultures may be encouraged to value interdependence, responsibility and cooperation, whilst learners within individualistic cultures may be encouraged to place higher value on personhood, rights, initiative and independence [24]. Hoffman [25] has critiqued the dominance of the Western cultural script within SEL research, and Dobia and Roffey [13] have noted that dominant cultural groups can misread the emotional expression of others and interpret them as deficient.

Dobia and Roffey [13] have, therefore, cautioned against simply providing school SEL programs that were designed to be effective with a general population to Australian Aboriginal students without considering and responding to the ways in which cultural differences in social and emotional wellbeing development and expression are understood [8]. For example, connectedness is a central theme within Australian Aboriginal definitions of social and emotional wellbeing [15,26,27,28], and Australian Aboriginal perspectives favor relational and collective understandings of wellness [29] over more individualized models, which are presumed in Western traditions. Connections to traditional country, cultural heritage, and language, profoundly influence the sense of identity and wellbeing for many Aboriginal Australians [15,26,27,28].

Within this collectivist understanding of identity, connectedness is more than the individualized sense of belonging to school and family, which has been identified as a protective factor for young people living in Western contexts [30]. Gee et al. [28] identified the interconnected domains of connectedness to body, connectedness to mind and emotions, connectedness to family and kinship, and connectedness to community, culture, spirituality, and country as critically underpinning the Aboriginal conceptualization of social and emotional wellbeing. Research specific to the Western desert Aboriginal people shows an emphasis on connectedness and relatedness in safeguarding the wellbeing of Aboriginal young men through, for example, provision of authoritative guidance, coupled with support and nurturance [31,32].

Although boarding schools have long been a part of the Australian education system, there has been little research into the use of SEL programs as support structures for boarding students generally, and even less for Aboriginal boarding students in particular [33,34]. Moreover, the little research that is available is inconsistent in its findings. Mander, Lester and Cross [35] analyzed social and emotional wellbeing data from 3462 students in the Catholic school sector before and after their transition from primary to secondary school. They found that the 150 boarding students in the study experienced greater levels of depression, anxiety and stress than non–boarding students. Contrary findings arose from a covariate analysis of the impact of boarding status on 5276 students, attending 12 Australian high schools [36]. The study investigated students’ motivation, engagement and psychological wellbeing, and generally found parity between boarding and day students, although some wellbeing outcomes favored the boarders. It is important to note, however, that both studies focused on the general population of students, with Martin and colleagues reporting that only 5% of their respondents identified as Aboriginal [35,36].

Few studies have specifically considered SEL programs for Aboriginal boarding students. Redman-Maclaren and colleagues [37] reported baseline findings from a study of the change in resilience and wellbeing in a group of 94 Queensland Aboriginal students at different stages of their education. They found that most of the primary school students reported high levels of resilience, but those attending secondary boarding schools reported lower levels. In addition, the psycho-social wellbeing scores of these secondary school boarders were less than for students still at primary school. A study by Bobongie [38] explored the boarding school transition experiences of girls from the Torres Strait Islands using a narrative enquiry approach. The experiences of the girls varied considerably, depending to a large degree on the support provided to them. Some of the girls were thankful for the opportunity provided by a boarding education. However, a common theme in the girls’ stories was that poor community support and a lack of cultural understanding within the school made the transition more difficult.

Mander [39] investigated the boarding experience of Aboriginal Western Australian male students and their parents. He found that both students and parents viewed attending boarding school as an opportunity to succeed, despite the accompanying social and emotional distress. In order to succeed the Aboriginal boarders needed to overcome homesickness. They also needed to manage the challenges associated with navigating changing relationships with peers in home communities, experiencing being a cultural minority, code switching and managing academic expectations. Mander and Fieldhouse [40] reflected on the nature of programs needed to support Aboriginal students through their education journey. They indicated that no single strategy would likely be effective—rather, a number of co-ordinated strategies are needed. They suggested that the strategies should incorporate students’ understanding of what, in practical terms, will help them deal with the specific social and emotional issues they experience, such as homesickness, loneliness and peer conflict.

This review of research indicates the need for a SEL program designed to suit Aboriginal Australian boarding students. Wellbeing education strategies are more likely to be effective when they are responsive to the social and cultural world students live in, address the specific challenges they experience and integrate students’ perspectives on what is needed to address these challenges [13]. Research suggests that responsivness is best achieved by utilizing a two-way co-design approach to learning that involves exchanges of Aboriginal and non-Aboriginal knowledges [13,31]. Informed in this way, the learning activities need also to be designed so as to be sufficiently open ended for participants to identify the social and emotional challenges they wish to address and to contribute their own interpretations and solutions to the challenges under consideration. Accordingly, this study sought to investigate the effectiveness of the pilot ‘Making Connections’ SEL program that incorporated Aboriginal and non-Aboriginal knowledges in improving the SEWB of Aboriginal boarding students. A secondary aim was to identify contextual factors related to remote Indigenous communities and the boarding school environments (e.g., historical, natural, cultural, societal, spiritual/religious, economic, technological, political) that influence the acceptance and effectiveness of the program.

## 2. Materials and Methods

### 2.1. Study Design

A SEL program that was substantially designed to attune to the cultural and social context of Aboriginal boarding students from remote communities was evaluated using an explorative mixed-methods pilot study. Based on the philosophy of pragmatism, mixed methods research is a synthesis of quantitative and qualitative research. Pragmatism is a powerful third paradigm choice that delivers research results containing more detail and greater balance [41]. The qualitative component of the mixed methods methodology reduces the possibility of importing dominant culture constructs and research tools into research with more contextually nuanced Aboriginal cultures [42]. The research design combined a pre-post, quantitative evaluation of the student SEL program with post qualitative evaluation. In the qualitative component, two gender-specific student focus groups and individual episodic interviews with the teachers and boarding staff were conducted to gather data about the context in which the SEL program operated. The study was approved by the Charles Darwin Ethics Committee (H16055).

### 2.2. The ‘Making Connections’ Social and Emotional Learning (SEL) Program

The ‘Making Connections’ SEL program that this study evaluated is a 10-session program that drew on some elements of the Resilience, Rights and Respectful Relationships (RRRR) SEL program [43,44]. This program was chosen as a modified version had earlier been used with positive results in a classroom-based study involving all Year 7 and 8 students from the same school [45]. The RRRR program draws on a heritage of thinking from both sociology and psychology. It features the use of collaborative learning and critical thinking activities designed to develop emotional literacy, character strengths, problem-solving, positive coping and help-seeking as well as positive gender relationships [46]. This program uses participatory learning strategies to activate student voice and engage students in collaborative approaches to critical thinking and problem-solving.

The program was substantially re-designed to attune to the context and needs of Aboriginal boarding students. The revised program drew upon the holistic Aboriginal conceptualization of wellbeing as suggested by Gee et al. [28], which emphasizes the interconnected mental, physical, cultural and spiritual aspects of health, family and community, and recognizes the impacts of intergenerational trauma due to historical and contemporary colonization [31]. The program employed a playful and participatory mode to attune to the delivery in an after-school context. The program also made central use of participatory games to open and close each session with yarning circles in which students were invited to call upon and share their cultural knowledges and strengths as a reference point against which to consider challenge and coping responses.

Furthermore, the program provided scenarios informed by Mander’s research [39], which raised issues such as loneliness, social isolation and separation in the boarding school setting, along with challenges in managing peer relations, schoolwork pressures, transitions to and from school and community, and family worries. Students devised additional scenarios to ensure local relevance and chose which scenarios they wished to discuss during small group activities. A range of activities involved students in identifying what helps them cope with change and challenge, how they offer support to each other, and how they might engage in help-seeking or peer referral if concerned about a peer. The 10-session program was jointly facilitated by a male and female teacher or a male and female residential staff member in the early evening during the period normally devoted to homework. The sessions were delivered on occasion to single gender groups and at other times to mixed gender groups. The sessions averaged 1 hour in length but varied somewhat in order to cover the topic content. A content overview of the ‘Making Connections’ program lessons is outlined in Table 1.

The boarding staff and teachers delivering the program participated in two days of preparatory professional development in the ‘Making Connections’ Program. This training incorporated a summary of the evidence-base informing the program and active sampling of each of the session activities in the program. Emphasis was given to modelling and explicit coaching in use of the games and collaborative learning strategies. The training emphasized the importance of positive ‘holding’ relationships on the part of teaching and boarding staff, and the essential contribution of a positive relational climate within the learning group [31,32].

### 2.3. Study Participants

All boarding students from Years 7–9 (average ages 13–15 years) in one Australian Northern Territory school were recruited to participate in the study. Of the 28 students recruited (10 female, 11 male, 7 unidentified), 21 completed both pre and post surveys. All participating students came from remote Aboriginal communities. All students had actively consented to participate in the program, with accompanying permission from their parents or guardians/adults acting in loco parentis.

### 2.4. Pre-Post Student Surveys

The student survey was adapted from the instrument used in the earlier classroom based SEL intervention in the same school [45]. The language was modified to suit Aboriginal students and the survey focused on elements of social and emotional wellbeing more relevant to this group. Anonymity was maintained by using a student-generated code based on fragments of easily remembered personal information. This code allowed matching of individuals over the course of the study without knowing their identity.

The survey incorporated the Kessler 5 (K5) [47]. The K5 comprises a robust five question subset of the Kessler Psychological Distress Scale-10 (K10), which is a widely used screening tool for non-specific psychological distress [48,49]. Slight wording changes were made to two of the original Kessler items to enhance understanding in an Aboriginal context [47]. The K5 asks about recent symptomatic feelings of depression and anxiety rated on a 5-point scale from 1 (none of the time) to 5 (all of the time). For each question, 1 is the minimum score and 5 is the maximum score. The minimum aggregate score is 5 and the maximum score is 25. A score in the range 5–11 indicates low/moderate psychological distress and a score in the range 12–25 indicates high/very high psychological distress [48].

An abbreviated version of the Resilience and Youth Development Module (RYDM) from the California Healthy Kids Survey was incorporated in the survey instrument to measure students’ internal and external resilience factors that have been linked to positive developmental outcomes [50]. This instrument uses a multi-level approach to examining resilience and has been demonstrated to show conceptual adequacy [51]. Following on from the research by Midford et al. [45], items drawn from construct groupings considered valid by Furlong, Ritchey and O’Brennan [52] and items measuring immediate influence factors were incorporated in the survey. The selected items focused on the personal abilities of the students and the influence of their school environment.

Students were asked to rate statements on ‘Internal Assets’ (12 items in 4 subscales: Self-efficacy, Empathy, Problem Solving, and Self-awareness) and ‘School Resources’ (14 items in 3 subscales: School Support, Meaningful Participation, and School Connectedness). Response options ranged from 1 (not at all true) to 4 (very much true) on an unbalanced, 4-point Likert scale. Additional statements on ‘Boarding Group Connectedness’ (5 items) and ‘Social and Emotional Skills’ (5 items) were included in the survey on the basis that they measured specific issues taught in the SEL curriculum. These original items in the study’s survey are listed in Table 2. Response options ranged from 1 (strongly disagree) to 5 (strongly agree) on a balanced 5-point Likert scale. The two measures developed specifically for this study, Social and Emotional Skills and Boarding Group Connectedness, had adequate reliability for research purposes (Cronbach’s α, respectively, 0.694 and 0.855), giving confidence each measured the nominated behaviors.

As treated analyses of the K5 and the four internal and external resilience measures were conducted using SPSS v24 (IBM Corp., Armonk, NY, USA). Paired *t*-tests were used to assess whether there was a significant change in any of the measures between baseline and post-intervention.

### 2.5. Qualitative Post Focus Groups and Individual Episodic Interviews

The qualitative component utilized symbolic interactionism as the theoretical framework [53,54] and grounded theory as the methodology [55,56,57]. In line with grounded theory, theoretical sampling was used throughout the study. The data was collected using focus groups and episodic interviews [54]. Five girls and five boys from the respondent group, who the teachers and boarding staff identified as confident expressing themselves in English, agreed to participate in two gender specific, post intervention focus groups. A post intervention focus group was also conducted with the two teachers and two boarding staff (one male and one female of each) who had delivered the program.

The two student focus groups asked students what they learned from participating in the SEL program, what aspects they enjoyed and what aspects they disliked. The students were also asked to suggest how the program could be improved. The focus group with teachers and boarding staff asked about their experiences delivering the program. In particular, they were asked to identify the program elements that worked well and those that did not, and how the program could be better tailored to the needs of Aboriginal students from remote communities. The focus groups were conducted by two interviewers, for support, elaboration in questioning the respondents and to ensure a more complete record of responses. Written notes were taken in all focus groups, as prior discussion with school staff indicated recording of the interviews would be inappropriate. The notes taken by each researcher were typed and compared for consistency immediately after the focus groups.

In line with theoretical sampling, insights from focus groups were used to inform additional sampling to gather in-depth information from teachers and boarding staff using individual episodic interviews. These interviews averaged an hour in length and were conducted by one researcher to gather data on the psychological and contextual factors influencing program implementation and how they interact over time. Notes were immediately typed after each interview then returned to the interviewee for checking. All teachers and boarding staff elected to engage in the checking process [58] and returned their interview notes with minor amendments or elaborations.

The data was analyzed using grounded theory analysis strategies [55]. Analysis cycled between open, axial and selective coding as new data were added to conceptualize the psychological and contextual factors that in interaction influence the SEL program over time. Key issues of program engagement, utility and improvement were identified. ATLAS.ti (version 7) data analysis software (ATLAS.ti Scientific Software Development GmbH, Berlin, Germany) was utilized for its focus on coding procedures support of grounded theory analysis, and facilitation of rigor and data organization [59,60]. Triangulation was achieved by cross verification of qualitative and quantitative data, and discussion between researchers during data analysis to enrich understanding and increase validity [61].

## 3. Results

### 3.1. Pre-Post Student Surveys

The mean pre and post K5 and the four internal and external resilience measures are presented in Table 3, accompanied by standard deviations paired *t*-test values, degrees of freedom and *p* statistics. ‘School Resources’ was analyzed as two separate scales because some items used a 5-point Likert scale, while others used a 4-point scale.

The mean K5 score indicated the boarding students were experiencing high/very high levels of psychological distress at pre and post-intervention. After receiving the program, there was no significant improvement in student scores. Similarly, there was no significant change on the RYDM Internal Assets, RYDM School Resources (4-point scale), RYDM School Resources (5-point scale) and Boarding Group Connectedness measures. However, there was a significant change in the Social and Emotional Skills measure, with average item scores increasing from 2.99 at baseline (just under pretty much true) to 3.28 post-intervention (between pretty much true and very much true).

### 3.2. Qualitative post Focus Groups and Individual Episodic Interviews

Data collected from students and teachers/boarding staff about the SEL program were analyzed based on four major themes: (1) what was liked about the program, (2) what was disliked about the program, (3) social and emotional skills/outcomes of the program, and (4) suggestions for improvement. The key findings for each theme are presented below starting with the student focus group findings, followed by the teachers/boarding staff findings.

The students liked the practical nature of the program activities, preferred activities that were student led, and enjoyed participating in the participatory games. The students preferred it when the boarding staff rather than the teachers facilitated the program as they already had a more personal relationship with them. This pre-existing, more holistic relationship meant that engaging in program activities did not feel similar to schoolwork. Both male and female students preferred the gender-segregated sessions, as they felt less pressure to conform to role expectations, and were more relaxed and natural in their participation.

The preference for boarding staff over teachers as facilitators stemmed from not knowing the teachers as well and feeling they were expected to behave as students in a classroom, which inhibited their participation in activities. They did not like it when didactic teaching methods were used because it devalued their perspectives on the issues being considered. They did not like the program being delivered instead of their one-hour evening homework period, as by this time they were tired, hungry and often arrived late because of sporting commitments. They reported that some topics were boring because the material covered issues that they felt they already understood. They also said that the activities used too many words they did not understand, and involved too much reading and writing. The students felt more confident about participating when the activities were more talk based.

Overall, students thought it was a good program for discussing the social and emotional issues they faced, and for providing them with the opportunity to build practical skills in this area. They said they developed greater self-confidence, which made them more comfortable expressing their opinion and helping others experiencing difficulties. The students believed they developed better skills for managing conflict and working with others in group situations. They also indicated the program improved their ability to concentrate when in class.

In terms of improving the program, the students suggested the sessions be delivered several times during the week rather than just once, as it would be easier for them to remember and build on what had been learned previously. They suggested the same boarding staff, who they knew, deliver the program as they already had a relationship with them and were more comfortable talking about emotional issues with them. The students wanted the program to focus more on helping them develop emotional skills, including developing personal skills that would make it easier to get a job when they left school. Finally, the students suggested the program provide more opportunities for cultural sharing as they missed that when they were away from home.

The analysis of the teacher and boarding staff focus groups and individual episodic interviews shows that the teachers and boarding staff valued the training they received to implement the program. They considered the activities practical, well-structured and with a good explanation as to relevance for students. They also valued being provided with the theory that underpinned the program activities and the research evidence that supported their effectiveness. Both groups agreed that the students responded better when boarding staff took the lead in facilitating activities because of the more relaxed nature of the relationship, which confirms the perspectives of the students. Also like the students, the teachers and boarding staff found that the gender-segregated sessions were better managed by the students and more productive in terms of topic focus.

The teachers and boarding staff indicated that the interactive and animated nature of the program coupled with the variety of activities was engaging for the students. The use of games was mentioned specifically as setting the right tone for the sessions, which was further enhanced if a non-classroom setting was used as the workshop space. The teachers also mentioned that their prior involvement in delivering the SEL program to all Year 7 and 8 students in the school was a considerable advantage in terms of enhancing their confidence using student centered interactive techniques.

One teacher made the point that it was hard for the students to relate to teachers as anything other than as authority figures who expected them to behave well and focus on their study. This situation tended to make teacher-facilitated sessions default to the instructive pattern of a classroom lesson, even though that was not the intention or design of the program. Didactic delivery also tended to occur when teachers prioritized ‘covering the material’ in the allocated time. Both groups indicated that the co-gendered sessions were problematic because the students were distracted and found it hard to focus on the topic. Delivery of the program in the evenings was not considered ideal because the students were running out of energy.

There was also general agreement that the program sessions should be grouped closer together to aid memory and continuity as the weekly interval between sessions meant time had to be spent reviewing what had been covered in the previous week. Another issue mentioned by the teachers and boarding staff was that some students, particularly boys, could not see the point of the non-competitive nature of the collective games played because there were no winners. Finally, both student and facilitators made the point that the literacy requirements of the program could be better tailored to the students and should contain more culturally relevant material.

The teachers and boarding staff agreed that throughout the program there was a noticeable improvement in student social and emotional skills. They particularly identified improvements in the students’ ability to speak in public situations, to seek help from adults and to work co-operatively with other students. They also commented on the personal growth they observed in the students over the course of the program. For their part, students said they were more confident about sharing their cultural background and showed greater cross-cultural understanding.

There was range of suggestions from teachers and boarding staff for improving the program. As a general approach, seeking ideas from senior boarding students was seen as giving greater relevance and substance to the program because of the challenges they had experienced and the skills they had developed to overcome them. In terms of program content, the suggestions stressed less reliance on literacy skills; greater emphasis on identifying and reinforcing existing strengths; acknowledging and rewarding task completion; and maintaining a focus on emotional regulation, conflict resolution, relationship skills and strategies for maintaining a sense of wellbeing. In terms of program delivery, the suggestions emphasized that the pedagogy should be informal, interactive, affirming and enjoyable; games should be included, but with a purpose understood by students; the sessions should be delivered during the day, rather than in the evening and should be grouped close together. Small, gender-specific groups are preferable; and there should be activities that facilitate cultural sharing. In terms of program facilitation, there was strong support for the program being delivered by boarding staff who were familiar to the students and with whom students had pre-existing relationships. An additional suggestion was to have a respected Aboriginal facilitator where possible.

## 4. Discussion

The primary aim of this study was to evaluate the relevance and effectiveness of a pilot SEL program that had been designed for use in the Aboriginal boarding context. The quantitative evaluation produced mixed results. The social and emotional skills of the students did improve significantly from pre to post intervention, but there was no significant change on the K5 Psychological Distress, ‘Internal Assets’, ‘School Resources’ and ‘Boarding Group Connectedness’ measures. The qualitative data about program relevance and contribution was more affirming. The analysis of the three focus groups (male student, female student and teachers/boarding staff) and the individual episodic interviews with staff indicated that both students and staff consistently and independently reported an increase in student social and emotional skills such as seeking help from and giving help to others, working in groups, managing conflict and asserting themselves. In addition, the students themselves experienced, and staff observed, the following associated outcomes: increased self-confidence, better ability to focus on school-work, and personal growth in general. Interestingly, and importantly, the teacher/boarding staff identified greater student confidence in discussing cultural issues. The qualitative findings also provided insights into what worked, what did not work and how the program could be improved.

These mixed results are encouraging in the context of what can be realistically achieved in the short term by a pilot SEL program in its early phase of development and testing. The qualitative results are consistent with the considerable research on the benefits of SEL programs [1,19,62] and should be seen as an early indication of the capacity of SEL programs to improve the SEWB of Aboriginal boarding students. This is also likely the case for the significant improvement in the quantitative measure of student social and emotional skills as Sklad and colleagues [22] reported that SEL programs had the greatest and most immediate effect on behavioral skill outcomes compared to more derivative outcomes such as mental health. The lack of significant change on measures of psychological distress, and internal and external assets, may be due to outcomes occurring as a consequence of student behavior and thus taking time to develop [63]. Accordingly, it is not surprising that there was no significant change on these measures at post data collection as the evaluation closely followed program delivery, allowing little time for the development of change.

While the changes associated with the program are encouraging, the results from the K5 measure of psychological distress are a cause for concern as they indicate that the boarding students were experiencing high/very high levels of psychological distress at both times of measurement. These results are congruent with research evidence of high psychological distress and heightened mental health risk among Aboriginal young people [9,11,12,13]. These results also add to the argument for additional research into the provision of culturally appropriate SEL programs for Aboriginal boarding students.

Two strong themes emerged from the focus group data that relate to the secondary aim of this study, namely identifying the contextual factors that influenced the impact of the program. The first key theme is connection or relatedness. The relationship between the boarding students and boarding staff emerged as critical for facilitating students in engaging in the program. Insights into the importance of a positive relational climate are also provided by LaGuardia and Ryan [64] and Reeve, Deci and Ryan [65]. These researchers reported that students learn better when they have a strong sense of relatedness as this enables them to take on challenges, internalize social regulations, have positive expectations and adapt to changing interpersonal circumstances. Similarly, findings emerged from a meta-analysis of 99 studies investigating the influence of teacher-student relationships on positive affect, attainment and engagement in learning. This meta-analysis demonstrates that positive teacher-student relationships are particularly important for adolescent students and for students deemed by their teachers to be vulnerable or marginalized in the school setting and at risk of disconnection from school [66].

However, students reported that they prefer to engage in SEL with boarding staff rather than teachers because the relationship with boarding staff is more personal and open, and occurring in an informal setting that is being less constrained by authority and didactic teaching. In such relationships there is a sense of safety that provide a secure base from which to learn. The importance of the teacher student relationship, specifically in the Aboriginal context, was also outlined Dobia and Roffey [13] in their research on the Aboriginal Girls Circle SEL intervention. They found that as openness and connectedness grew among students, and between students and the project officer, a sense of cultural safety became apparent in which students did not feel judged and could safely share their experiences. The finding of connection or relatedness as central to facilitating student engagement with the SEL program reinforces existing research on the importance of connectedness in underpinning the Aboriginal conceptualization of SEWB [15,26,27,28], and emphasizes the importance of using these conceptualizations of SEWB in SEL program design and delivery.

The boarding staff interacting at a personal level with the students, outside of the formal education context, enables them to better able to balance authority with acceptance and nurturance in ways to which young Aboriginal people can relate. When Aboriginal students come to boarding school, they face a loss of the emotional security that comes from living with their family, having an extended network of kinship support and understanding their Aboriginal cultural environment. At the same time, they need to adapt to considerable changes in their education process and cultural setting, while experiencing the challenges of adolescence. Experiencing trusting and nurturing relationships with boarding staff would seem to be central to improving SEWB in the boarding setting. Hence, creating a secure relational base within the boarding school environment is an important contextual factor influencing the contribution of a SEL program.

The second contextual factor was quality and consistency of program delivery, which when compromised was disruptive and negatively affected engagement and security of the students. There were three key inconsistencies reported that affected the quality of program delivery. Firstly, the program was not consistently led by the same facilitator(s) due to staff rostering. This inconsistency was reported by the students as making it more difficult to create trusting relationships and a safe learning environment. This factor was heightened due to the disparity in the more formal facilitation style used by the teachers and the more relaxed style of the boarding staff. A second factor disrupting the relational process was the presence of observers in a number of sessions. These observers were visiting educators not known to the students who attended to witness the program in operation as it was a novel initiative within the school. These interruptions inhibited student participation and interrupted the natural flow of the sessions.

A third inconsistency in program delivery occurred in relation to group membership. On some occasions the sessions were delivered to mixed gender groups, while in other instances the sessions were conducted in single gender groups. The group membership depended on the topic being presented as some topics were gender sensitive and it would have been culturally inappropriate to include both genders. There was also trialing of the type of group that worked best in terms of student engagement. Feedback from all focus groups indicated that the single gender groups worked best overall and exploration of this issue will usefully inform future SEL programs with Aboriginal boarders. However, in the context of this study the discovery process introduced inconsistencies that were disruptive.

This study is limited by the small number of participating students from one school and by the absence of a control group. However, the study was designed as a pilot for the purpose of exploring the relevance and contribution of a SEL program in its first phase of piloting with Aboriginal boarding students. Desired outcomes of the pilot included suitability of the study design, evidence of program effect, and student and facilitator advice for program development. Consequently, the design limitations have to be balanced against the benefit that can come from a small pilot. The scope of the focus groups with students was also limited by the scale of the study. Only one focus group was conducted with each gender and the students were invited based on their ability to comfortably express themselves in English. Accordingly, the views expressed came predominantly from older students who were more confident in using English as a language to express their views, meaning that the perspectives of younger students and those with more limited English were not included.

## 5. Conclusions

This study has demonstrated that an evidence-informed, culturally responsive SEL program designed for Aboriginal middle school students (Years 7–9) can be effectively delivered by boarding staff in the boarding school setting, can achieve student endorsement, and can lead to significant improvement in student social and emotional skills. Particularly important findings for the context and delivery that underpinned engagement with the program was the need for secure relationships with those delivering the program, the importance of cultural sensitivity as an enabler of effective engagement, and the importance of adhering to participatory rather than didactic modes of program delivery. Secure relationships with facilitators hold students in a safe and nurturing space, and these secure relationships made it easier for students to explore cultural, social and emotional issues together. Accordingly, future SEL programs for Aboriginal boarders need to consider not just program content and delivery, but additionally the choice of facilitators to ensure program provision occurs in the context of secure, authoritative and nurturing relationships. The finding that complex and changing contextual factors, especially cultural factors, affect the relevance and impact of the program lends further evidence to the importance of co-designing, co-evaluating, co-facilitating and co-revising programs together with Aboriginal peoples [67]. As student recommendations also emphasized the need for greater exchange and development of cultural knowledges, future program development should pay greater attention to drawing on cultural understandings of SEWB to inform the design and facilitation of program learning activities, should ensure that participatory activities structure opportunities share cultural knowledge, and that organizational arrangements support consistent delivery and relational support.

## Figures and Tables

**Table 1:** Overview of the ‘Making Connections’ program lessons.

Lesson	Content
1. Connections to feelings	Introductions, emotions and their triggers. Using games for teamwork skills.
2. Connections to Friends	Fostering positive peer relationships by investigating unequal power dynamics in relationships, the experience of multiple or mixed emotions, and hidden emotions (emotional layering). A focus on friendship and peer support via acts of kindness.
3. Connections to strengths from People and Country	Valuing and sharing the strengths learnt from my people and culture. Exploring leadership through cultural role-models. Considering the strengths of trust and courage in peer support and help-seeking.
4. Connections to strengths in friendship	Sharing strengths learnt from my relationship with country. Drawing on strengths when dealing everyday challenges. Strength of teamwork in friendship. Empathy. Connecting valued strengths from country to actions in friendships.
5. Connections to strong ways of coping	Talking about helpful and harmful coping strategies. Sharing productive coping strategies for use in different situations. Using coping strategies to deal with homesickness. Sharing about how to use music to cheer up or to calm down.
6. Connections to clear mind in choices	Focusing on problem-solving strategies to use in relation to common scenarios. Skills for cooperation and communication in problem-solving with peers.
7. Connections to the coach in the head	How to use positive self-talk when dealing with negative thoughts, emotions and events. Using cultural and personal strengths for positive self-talk.
8. Connections to calm mind and body	Sharing stress management and self-calming techniques, such as muscle relaxation, breathing techniques, exercise and listening to music.
9. Connections for friends in hard times	Talking about how to provide peer support when people are in tough times. Thinking through when to call on adults to help support troubled peers.
10. Connections to help in tough times	Practicing help-seeking conversations for self and others. Identifying supports to turn to for help. Feedback and advice for improving the program.

**Table 2:** Boarding group connectedness and social and emotional skills items.

Measures	Items
Boarding Group Connectedness	We do good activities during evening study
	People in my evening study group care about each other
	I can get along well with others in my evening study group
	I feel like my evening study teacher and house parent know me well
	I am close to other kids in the boarding house
Social and Emotional Skills	When I need help, I find someone to talk with
	I have ways to calm myself down when I feel stressed
	I know I have some strengths
	I can think of good strategies to help with different sorts of challenges
	I have ways to cheer myself up when I feel down

**Table 3:** Student survey component scores, pre and post intervention.

Measure	*n*	Pre-Intervention		Post-Intervention		*t*	df	*p*
		**Mean**	**SD**	**Mean**	***SD***			
K5 Psychological Distress	20	15.70	4.80	14.00	4.18	1.73	19	0.100
Internal Assets	20	37.00	6.55	37.50	6.75	−0.41	19	0.685
School Resources	21	27.48	5.52	28.62	5.35	−1.44	20	0.167
School Resources	21	19.52	3.97	19.67	3.97	−0.16	20	0.878
Boarding Group Connectedness	21	19.00	4.24	20.43	3.83	−1.32	20	0.201
Social and Emotional Skills	20	14.95	3.17	16.40	2.87	−2.66	19	0.015 *

*Note:* A total of 21 students provided pre- and post-intervention data, however, data was missing on some measures for two students. *: *p* < 0.05.
